# Perioperative poor grip strength recovery is associated with 30-day complication rate after cardiac surgery discharge in middle-aged and older adults - a prospective observational study

**DOI:** 10.1186/s12872-019-1241-x

**Published:** 2019-11-27

**Authors:** Liyuan Fu, Yuanyuan Zhang, Bohan Shao, Xiangjing Liu, Bo Yuan, Zhengqing Wang, Tienan Chen, Zhigang Liu, Xiaocheng Liu, Qi Guo

**Affiliations:** 1 Department of Rehabilitation Medicine, TEDA International Cardiovascular Hospital, Cardiovascular Clinical College of Tianjin Medical University, Tianjin, China; 2 Department of Rehabilitation Medicine, Tianjin Medical University, Tianjin, China; 3 Department of Rehabilitation Medicine, Shanghai University of Medicine and Health Sciences, Shanghai, China; 4 College of Rehabilitation Sciences, Shanghai University of Medicine and Health Sciences, 279 Zhouzhu Highway, Pudong New Area, Shanghai, 201318 China

**Keywords:** Cardiac surgery, Complication, Early stage, Grip recovery

## Abstract

**Background:**

Although perioperative care during heart surgery has improved considerably, the rate of postoperative complications has remained stable. It has not been concluded how to better apply grip strength to clinical, postoperative complications. So our study aimed at researching the best way for using grip value for predicting early postoperative complications.

**Methods:**

A total of 212 patients with mean age 63.8 ± 6.3 who underwent cardiac surgery participated in our study. We analyzed the ROC curve of grip strength, grip/weight and grip recovery with complications, found the best cutoff point. Logistic regression confirmed the association between grip strength grouping and complications.

**Results:**

We found that 36 patients had 30-day complications. EuroSCORE were 2.15 ± 1.52 and 2.42 ± 1.58 between normal and complication groups, respectively. The area under the receiver-operating characteristic curve (AUC) of grip recovery take the most area (0.837, *p* < 0.001), and the cutoff point was 83.92%. In logistic regression, lower grip recovery has higher risk impact on 30-day complications for 25.68 times than normal group, after adjusted surgery-related factors. After regrouped characteristic information by grip recovery cutoff point**,** we found that percentage of the estimated 6 min walk distance (41.5 vs 48.3, *p* = 0.028) and hospitalization time (7.2 vs 6.1, *p* = 0.042) had worse trends in lower recovery group.

**Conclusions:**

Poor grip recovery may be related to higher risk of postoperative complications within 30 days after discharge in middle-aged and older people independent of surgical risk. The results of this study provide a reference for the development of rehabilitation programs in the early postoperative recovery, and may also be a prognostic indicator for postoperative high-risk groups.

**Trial registration:**

Our research was registered on Research Registry website, the registry number was ChiCTR1800018465. Date: 2018/9/20. Status: Successful.

## Background

Although perioperative care during heart surgery has improved considerably [1–3], the rate of postoperative complications has remained stable [4, 5]. It is well known that prolonged complication-related hospitalizations and readmissions after initial hospitalizations are frequent, especially 30 days after hospital discharge. It can significantly affect clinical outcomes and patient quality of life by leading to longer hospitalization times and more health care costs [6, 7]. Therefore, it is very important to find out the factors related to postoperative complications from cardiac surgery and to find corresponding prevention or treatment measures. Recently, postoperative outcome at 30 days has been widely recognized as an important early stage in postoperative recovery [8–10], especially for cardiac surgery [11–15]. Middle-aged and older people, who are more likely to be frail, have been reported to have inferior surgical outcomes [16, 17] and an elevated risk of postoperative complications [1]. Patients with limited ability to recover following heart surgery are more likely to have postoperative complications, increasing length of hospital stay and overall resource utilization.

As one of physical functions related to frailty, muscle strength is negatively correlated with all-cause mortality, cardiovascular mortality independently of cardiac-related factors [18], and it is an important indicator of cardiovascular disease. Preoperative physical performance, especially grip strength, was related to some traditional assessments (i.e. ejection fraction and lung function) which was shown to be associated with cardiovascular mortality [19, 20], but recently the available study showed that different forms of grip strength may have a stronger correlation with prediction for the evaluation of cardiac surgery prognosis. Another better indicator might also exist. One study investigated the impact of weight loss and functional status on outcomes after coronary artery bypass grafting (CABG) showed that grip strength decreased in those patients who developed complications [14]. The result of this study, however, has not been widely accepted. So it is important to determine how to better use grip values for predicting complications in clinical diagnoses. Our previous research showed there were no significant differences in the relationships between the absolute value of grip (preoperative and postoperative values) and postoperative complications. Recently, one study indicated that body weight fluctuations were associated with higher mortality rates and higher rates of cardiovascular events were independent of traditional cardiovascular risk factors [21]. Thus, we have reason to believe that changes in pre- and postoperative grip strength may be a better indicator of prognosis and rehabilitation targets than absolute values.

Therefore, the purpose of our study was researching whether grip recoveries could be a better indicator than grip strength and grip/weight at predicting postoperative complications 30 days after hospital discharge while excluding the effects of surgery-related factors. Meanwhile, we tried to find the cut point value (i.e. the diagnostic standard value) and observe the effects of the new diagnostic standard value. This might provide a reference point and important values for recovery and early rehabilitation after cardiac surgery. Postoperative complications of 30 days after hospital discharge might be an indicator of long-term prognosis.

## Method

We conducted a prospective trial to determine whether grip recovery, which was defined as postoperative value/preoperative value, could be a better indicator than grip strength and grip/weight at predicting postoperative complications 30 days after hospital discharge. The participants were fully informed of research nature and signing an informed consent form to participate. This study was approved by the Tianjin Medical University ethics committee.

### Participants

The trial was performed at a cardiovascular hospital in China. Our research was conducted from October (2018) to January (2019). During this time, we enrolled patients and recorded their information at baseline. Then we followed up on complications at 30 days after discharge in postoperative patients. Patients, who were scheduled for primary elective cardiac surgeries (including coronary artery bypass graft—CABG, valve replacement, other cardiac surgeries except for aortic dissection), and who had the ability to provide informed consent, were eligible. Exclusion criteria were: 1) age < 50 years old, 2) vision impairment without corrective lenses at the time of the tests, 3) diabetic neuropathy involving the hands, 4) refusal to participate in our study follow-up, 5) surgery-related bone trauma and deformation of the hand joints (i.e. surgical diseases affecting muscle strength and grip strength), 6) history of stroke, and 7) undergoing a repeat operation. The methodological sessions were carried out in accordance with the approved guidelines and regulations.

During the study period, 271 patients participated and underwent surgery. Of these people, 237 met the requirements, and 34 patients were lost to follow-up or had incomplete data. In the end, 212 patients were included in this study, and 69.3% of them were male. The average age of this population was 64.5 ± 6.1 years old. Among the patients, 134 patients underwent CABG surgery, 12 patients underwent aortic valve replacement surgery, 39 underwent non-aortic valve replacement surgery, and 27 patients underwent other surgeries. All the operations were performed by the same team. All surgeons had at least 5 years of experience.

### Preoperative and perioperative assessments

Demographics and preoperative factors were prospectively recorded during a standardized interview. Preoperative sociodemographic variables, including age, gender, weight, height, body mass index (calculated as weight in kilograms divided by height in meters squared), marital status, educational level, and occupation were assessed. Marital status was classified as married or not married/single. Educational level was defined as age at completion of schooling and divided into 4 categories: < 1 yr of schooling, 1–6 y, 7–12 y, and ≥ 13 y. Behavioral characteristics included smoking and drinking habits. Information on smoking (never, former smoker, or current smoker) and drinking (never, former drinker, occasional drinker, or everyday drinker) were also obtained from the questionnaire. Physical activity was assessed using the short form of the International Physical Activity Questionnaire (IPAQ). History of myocardial infarction, hypertension and diabetes mellitus were recorded from the medical records. Type of surgical procedure, current diagnoses, pulmonary status, duration of surgery, duration of Intensive Care Unit (ICU) stay, and duration of mechanical ventilation were also recorded. All surgery-related information was reported by the computer record. We used EuroSCORE to evaluate the surgical risk of patients and adjusted for it in our logistic models. EuroSCORE has been widely used for evaluating operative risk, and its validity and reliability have been verified for cardiac surgery [22, 23].

Performance-based assessments consisted of several physical tests. We had described the methods for gait function and grip strength in detail in our previous study, we added this detail to Additional file 1 [24]. Gait function was assessed with the 4-m walk test. To measure walking speed, two groups of recording laser transmitter receiver timers were placed at the beginning and the end of a 4-m course. Patients were asked to cover a distance of 4-m distance at the usual uniform speed. Grip (kg) was used as a measure of muscle strength and quantified using a handheld dynamometer (GRIP-D; Takei Ltd., Niigata, Japan). Participants were asked to exert their maximum effort twice by using their dominant hand, and the average value of grip strength was recorded. This method of standardization had been previously recommended in order to normalize and improve physical function testing results [25]. To avoid measurement error, the assessment was conducted by postgraduate students in the health field who received special training for administering all tests. Every project was carried out by one trained staff member to complete the data collection of all the subjects.

### Measurement of recovery

On the fifth postoperative day, we assessed postoperative patients by monitoring heart rate and blood pressure during a 6 min walk distance (6MWD) test. We also recorded the distance they could walk in 6 min. We calculated the percentage of the estimated 6MWD. Previous studies had verified the reliability and validity of the 6MWD for evaluating heart disease [26]. In order to measure postoperative hospitalization time, we also recorded the patient’s surgery date and hospital discharge date and then calculated the interval in days.

### Grip strength recovery

**Fig. 1:**
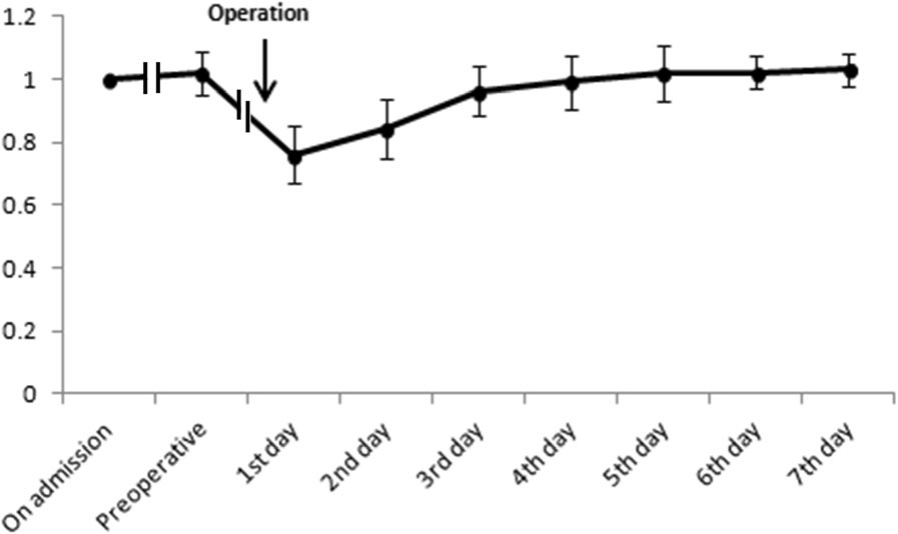
The trends of grip strength recovery after surgery

Grip strength was measured during the preoperative and postoperative period (i.e. fifth day after surgery). Before this study we had carried out a preliminary experiment and had found that on the fifth day after surgery patients were able to return to their preoperative walking abilities and that their grip strength plateaued. Figure 1 shows the relative value of grip strength on admission, preoperatively, and 7 days after surgery. We found that the rate of grip strength recovery becomes smoother on the fifth, sixth, and seventh days. Thus, we measured grip strength on the fifth day to represent average grip strength value. Grip recovery was defined as postoperative value/preoperative value. To determine the best predictors, we analyzed the relationship between grip strength, grip strength/body weight, grip recovery, and complications.

### Postoperative follow-up and definition of complications

Follow-up occurred at 30 days after hospital discharge. Patients were asked to undergo postoperative cardiac ultrasounds, chest radiographs, routine blood tests, etc. Complications were defined as death, needing for reoperation, atrial fibrillation, deep sternal infection, pulmonary complications, stroke, sensory changes, renal failure requiring treatment, dehydration, multisystem organ failure, and readmission to the hospital within 30 days [14, 15]. Complications during follow-up did not include those that occurred during the perioperative period or postoperative complications that occurred during hospitalization immediately after the surgery. All results were obtained at outpatient care appointments at our hospital or a field hospital. All reexamination results and complications were reviewed by medical staff. Patients without reexamination information were reached by phone for feedback. Missing follow-up data were excluded. Thirty-six patients had 30-day complications, and 176 people were normal.

### Statistical analysis

Differences between continuous variables were examined using t-tests with the Bonferroni correction. We used the chi square test on categorical variables. Data was presented as means and standard deviation or as percentages. Table 1 shows characteristics of patients with or without 30 day complications. Receiver-operating characteristic (ROC) curve analyses were performed to determine the optimal cutoff values for grip strength, grip/weight and grip recovery with complications as the outcome variable (Table 2). The receiver-operating characteristic curve was a graph of sensitivity plotted against (1 − specificity) over all possible diagnostic cutoff values. The optimal cutoff values were obtained from the maximal Youden’s index, calculated as (sensitivity + specificity − 1), and then the best combination of sensitivity and specificity was chosen. Logistic analysis was used to assess the relationship between optimal grip factors and complications 30 days after hospital discharge (Table 3). Covariates were added sequentially to the model to evaluate associations at different levels of adjustment. Crude analysis was unadjusted. Multivariable adjusted model 1 was adjusted for age, gender, and body mass index (BMI). To exclude the impact of surgery related-complications on grip strength and other complications, multivariable adjusted model 2 was adjusted for age, gender, BMI, EuroSCORE, smoking, drinking, hypertension, diabetes, hyperlipidemia, surgery duration, length of ICU stay, assisted ventilation time, and drainage time. In order to verify that grip recovery was the best predictor, we regrouped characteristic information by grip recovery cutoff point to observe postoperative correlation factors (Table 4). Values under 83.92% were defined as predicting a low chance of recovery. Differences were defined as significant when *P* < 0.05. All statistical analyses were performed using SPSS V19.0 software package (SPSS Inc., China).

**Table 1:** Characteristics of the Patients in groups with or without complications

Characteristic	Normal (*n* = 176)	Complication (*n* = 36)	*P*
General characteristic			
Men (n,%)	68.3	65.4	0.746
Age (y)	63.2 ± 6.6	64.4 ± 6.0	0.408
BMI (kg/m^2^)	25.6 ± 3.2	25.6 ± 3.1	0.435
Widow (%)	8.2	3.8	0.390
Farmer (%)	37.3	25.6	0.358
Live alone (%)	10	6.5	0.462
EF (%)	55.3	57.9	0.158
IPAQ	6994	6402	0.656
Drink (%)	52.8	52.9	0.368
Smoke (%)	32.9	38.5	0.089
Grip (kg)	30.8 ± 9.1	31.2 ± 10.5	0.255
Grip/Weight (kg/kg)	0.43 ± 0.11	0.45 ± 0.10	0.434
4-m walking(m/s)	0.97 ± 0.21	0.95 ± 0.21	0.808
EuroSCORE	2.15 ± 1.52	2.42 ± 1.58	0.235
Disease history			
Hypertension (%)	60.6	58.8	0.876
Diabetes (%)	25.4	30.6	0.478
Hyperlipidemia (%)	3.7	6.1	0.433
Coronary heart disease (%)	35.4	40.9	0.492
Operative situation			
Type of surgery			0.129
CABG (%)	71.9	70.6	
Aortic valve replacement (%)	12.7	18.6	
Non-aortic valve replacement (%)	7.2	5.8	
Other surgery	8.2	5.0	
Surgery time (h)	4.28 ± 1.78	4.67 ± 1.28	0.135
Length of ICU staying (h)	44.3 ± 13.6	51.2 ± 23.6	0.007
Assisted ventilation time (h)	12.2 ± 10.5	15.4 ± 15.6	0.094
Drainage time (h)	72.7 ± 46.7	89.7 ± 53.8	0.063
Recovery situation			
Grip fluctuation (%)	100.2 ± 35.5	65.3 ± 22.3	< 0.001
6MWD (m)	239.1 ± 102.1	204.8 ± 79.4	0.088
Percentage of the estimated 6MWD (%)	46.4 ± 19.3	39.8 ± 14.4	0.081
Heart rate recovery (%)	55.6 ± 23.3	54.4 ± 25.7	0.932
Length of hospitalization time (day)	6.4 ± 2.4	6.6 ± 2.9	0.418

BMI, body mass index; IPAQ, international physical activity questionnaire; EF, Left ventricular ejection fraction; 6MWD, 6 min walk distance

Mean ± Standard deviation in all such values

Obtained by using t-test for continuous variables and chi-square for variables of proportion

**Table 2:** AUC and cutoff point of complications and relative factors in patients

	AUC(95%CI)	*P* value	Cut off	Sensitivity (%)	Specificity (%)
Grip strength (kg)	0.457(0.355,0.559)	0.416	25.98	36.1	33.0
Grip/Weight (kg/kg)	0.476(0.372,0.581)	0.657	0.421	50.0	44.3
Grip fluctuation (%)	0.837(0.761,0.912)	< 0.001	83.92	80.6	49.8

*AUC* area under the receiver-operating characteristic curve, *CI* confidence interval

**Table 3:** Crude and multivariable-adjusted Odds Ratios using the cutoff values of grip fluctuation for 30 days complications

	OR	95%CI	*P* value
Grip fluctuation			
Crude	13.22	5.40–32.35	0.001
Multivariable-Adjusted^1^	13.49	5.47–33.29	0.001
Multivariable-Adjusted^2^	25.68	7.65–65.90	< 0.001

^1^Adjusted for age, gender, BMI

^2^Adjusted for age, gender, BMI, EuroSCORE, smoking, drinking, Hypertension, diabetes, hyperlipidemia, surgery duration, length of ICU staying, assisted ventilation time, drainage time

Obtained by using Logistic regression

*OR* odds ratio, *CI* confidence interval

**Table 4:** Characteristics of the Patients in groups divided by grip fluctuation cutoff points

Characteristic	Normal (*n* = 141)	Low recovery(*n* = 71)	*P*
Operative situation			
Surgery time (h)	4.18 ± 2.08	4.51 ± 1.28	0.232
Length of ICU staying (h)	44.5 ± 12.8	48.6 ± 22.2	0.114
Assisted ventilation time (h)	10.9 ± 7.2	16.4 ± 8.9	0.005
Drainage time (h)	74.7 ± 60.5	77.8 ± 52.9	0.744
Recovery situation			
6MWD (m)	248.5 ± 88.9	221.5 ± 109.2	0.097
Percentage of the estimated 6MWD (%)	48.3 ± 17.4	41.5 ± 18.9	0.028
Length of hospitalization time (day)	6.1 ± 2.8	7.2 ± 2.5	0.042
Complications (%)	5.0	40.8	< 0.001

*6MWD* 6 min walk distance

Mean ± Standard deviation in all such values

Obtained by using t-test for continuous variables and chi-square for variables of proportion

## Results

**Fig. 2:**
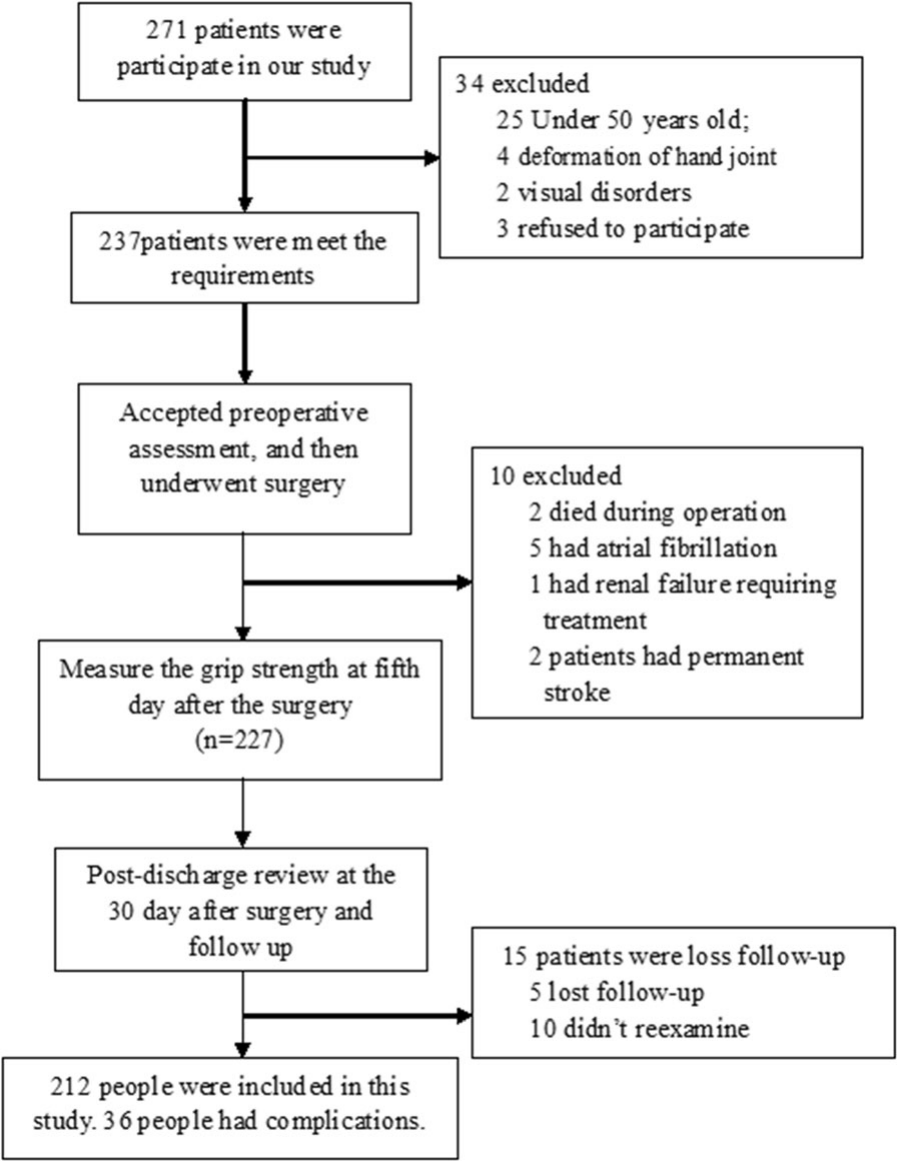
Our test flow chart

Among the 271 patients, there were 237 people met the requirements and included in this study. After a serial assessment and surgery, 10 patients were excluded, 2 patients died during the surgery, 5 patients had secondary atrial fibrillation during hospitalization, 1 patient had renal failure requiring hospitalization, and 2 patients had strokes during their hospitalizations. There were 227 people completing grip strength and 6MWD assessments on the fifth day after surgery and then discharging. The average length of hospital stay after surgery was 6.5 ± 2.7 days. Two hundred twelve came back on the 30th day after discharge and underwent ultrasounds, X-rays, and routine blood testing. Ten patients were not examining at either our hospital or at a local hospital, and five patients were lost to follow-up. After follow-up on the 30th day, we found that 36 patients had postoperative complications as shown in Fig. 2. Two patients died, three patients were newly diagnosed with atrial fibrillation, two patients needed reoperation, five patients had secondary stroke, thirteen patients had deep infections, and eleven patients were readmitting to the hospital.

We stopped follow up after operative 30 days. The characteristics of patients in groups with or without complications were shown in Table 1. The average ages of the two groups were 63.2 ± 6.6 and 64.4 ± 6.0 years, respectively. The EuroSCOREs in two groups were 2.15 ± 1.52 and 2.42 ± 1.58, respectively. Patients who underwent CABG surgery accounted for 71.9% of the normal group and 70.6% of the complications group, meaning CABG patients were majority. We found that only length of ICU stay was significantly different between the two groups (44.3 vs 51.2 h, *p* = 0.007), assisted ventilation time, drainage time, 6MWD results, and relative recovery situations were not significantly different. The average 6MWD in both groups were over 200 m. Heart rates 1 min after the completion of the 6MWD had returned to the original resting heart rates in more than 50% of all participants. The complication group showed a trend toward a longer hospital stay, but it was not significantly different from the group without complications.

**Fig. 3:**
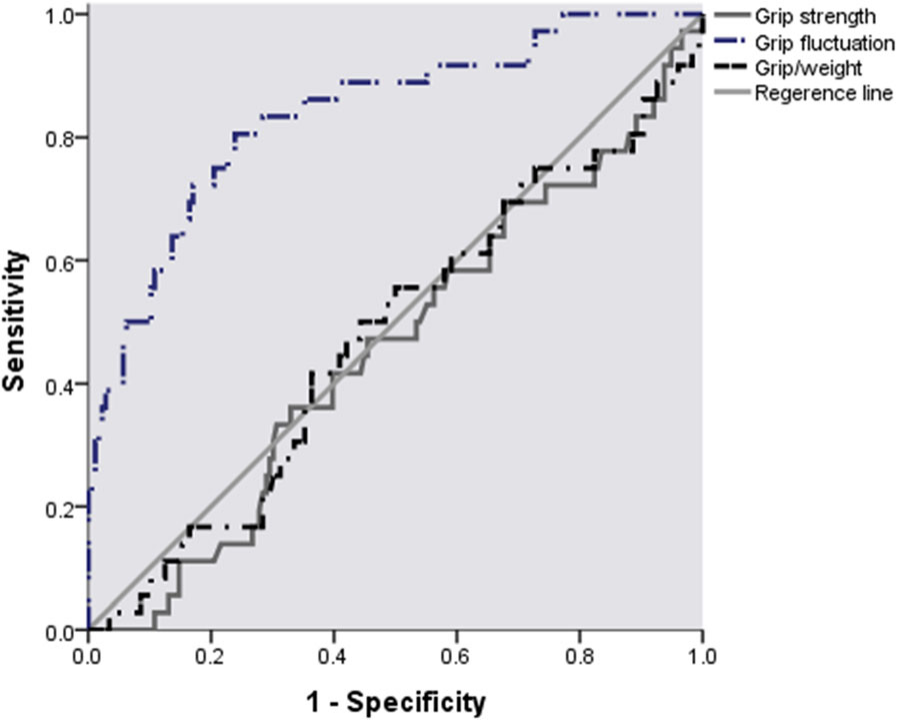
ROC curve of grip strength, grip recovery and grip/weight with complications

Then we conducted the study to determine which factor was most predictive of 30-day complications: grip strength, grip/weight, and grip recovery. We used ROC curves and found that the area under the receiver operating characteristic curve (AUC) of grip recovery had the most area (0.837, *p* < 0.001) with a cutoff point of 83.92% (Table 2, Fig. 3). The other two factors had areas less than 0.5, meaning they were not appropriate for predicting complications. Then we used the cutoff point to divide patients into two groups: the normal group had a grip recovery of more than 83.92% (*n* = 141), and the low recovery group (*n* = 71) had a grip recovery less than 83.92%. A logistic regression analysis was used to check whether lower grip recovery increased the risk for 30-day complications. The crude model showed that grip recovery was related to complications 13.22 (5.40–32.35). After adjusting for age, gender, BMI, smoking, drinking, hypertension, diabetes, hyperlipidemia, type of surgery, surgery duration, length of ICU stay, assisted ventilation time, and drainage time, the odds ratio (OR) for complications increased to 25.68 (7.65–65.90). Then we divided the participants into these two recovery groups and analyzed for differences in characteristic information, many postoperative factors were significantly different between these two groups. We found that lower recovery people had a lower estimated 6MWD percentage (41.5% vs. 48.3%), longer hospitalization times (7.2 days vs. 6.1 days), and longer assisted ventilation times (16.4 days vs. 10.9 days).

## Discussion

To our best knowledge, this study is the first investigation to address the relationship between grip recovery and postoperative complications at 30 days after hospital discharge. We note that except for length of ICU stay, type of surgery, surgery time, assisted ventilation time, and drainage time were not related to complications at 30 days (Table 1). To determine the best predictor of complications, we constructed an AUC curve, and found that grip recovery was the best factor when compared to grip strength and grip/weight values.

### The relation between grip recovery and postoperative complications

An abundance of evidence showed that grip strength was a strong predictor of cardiovascular mortality and a moderately strong predictor of incident cardiovascular disease. Muscle strength is not only a risk factor for incident cardiovascular disease but also a predictor of death risk in people who develop either cardiovascular or non-cardiovascular disease [18]. So the relationship between grip strength and cardiovascular disease is very important. Early studies showed that preoperative grip strength predicted postoperative complications during hospitalization in noncardiac surgical patients [27]. However, the absolute value of grip strength varies greatly due to age, gender and other factors, so the ability of grip strength to predict postoperative complications risk may be relatively inaccurate. Recently, one research showed that change in body weight before and after surgery was associated with elevated mortality and an elevated rate of cardiovascular events independent of traditional cardiovascular risk factors [21]. This indicates that the change in weight may be a predictor of cardiac disease. In addition, research involving abdominal surgery demonstrated a reduction in grip strength during the first week after surgery in older adults [28]. The researchers examined the change between grip strength prior to surgery and grip strength at 5 days after surgery. Our research referenced weight fluctuation research [21], and used grip strength, grip/weight, and grip recovery values to analyze the optimal factors for predicting complications. As expected, grip recovery was more predictive than absolute grip strength or grip/weight of complications. Furthermore, after adjusting for relative factors and post-surgery factors, we found that this group had an elevated risk of postoperative complications compared to the normal grip strength group. This result was similar to another prospective study [14], but this study measured grip strength at 4–6 weeks after discharge, which might explain the differences between the two studies. The grip strength measurement time point still needs to be confirmed.

### Potential factors in this relationship

There are many factors that affect grip recovery before and after cardiac surgery. Low preoperative skeletal muscle mass results in loss of muscle function in patients with cardiac surgery and higher mortality [29]. Muscle strength was related to health status. In addition, postoperative grip strength was associated with preoperative grip strength, as well as preoperative and post-discharge nutritional status [14]. This indicates that nutritional status could make a better postoperative grip strength recovery. Nutrition may also be an intervention for early postoperative rehabilitation. Operation-related factors are the main factors that influence grip recovery. We listed the surgery related factors in Table 1. We also found that duration of ICU stay, assisted ventilation, and drainage time were not related to low grip strength recovery after adjustments were made. It was suggested that poor postoperative grip recovery might have a higher risk for postoperative complications independent of operative situations. Grip strength can reflect overall muscle strength in the human body. Low grip strength recovery related to a reduced physical activity, a decline in respiratory muscle strength [30], an increased risk of falls, an increased occurrence of complications and readmission [31, 32]. Moreover, the extension of the inflammatory reparation process after cardiac surgery may also result in decreasing grip strength and therefore decreasing postoperative physical function [14]. Further study is needed to examine the influence of inflammation on postoperative recovery.

### Cutoff values for efficient clinical indexes

In the current study, receiver operating characteristic analysis showed that the optimal cutoff value for grip recovery was 83.92% and that this cutoff value could be used to target middle-aged and older adults who most likely would benefit from interventions preventing postoperative complications. The sensitivity of the grip recovery cutoff was 80.6%; however, the specificity was low (49.8%), implying that this cutoff would successfully predict people with complications but would also yield false positives 50.2% of the time. Due to the low specificity, optimal cutoff values should be used with caution. After comparing characteristics between the two patients groups divided by the grip recovery cutoff point, we found good trends in recovery situations regarding the percentage of the estimated 6MWD, length of hospitalization time, and complications, *p* < 0.05. These results further confirmed that the cutoff point was appropriate for assessing prognosis. Overall, this cutoff value has the potential to identify middle aged and older adults who might have postoperative complications. Grip strength reflects the whole body’s muscle strength. Low preoperative muscle strength could help doctors determine the risks of surgery and postoperative complications earlier. So it is important to start intense physiotherapy as early as possible to recover the grip strength. Further studies need to prove whether physiotherapy having effect on improving grip strength. This process should lead to an earlier recovery time as physiotherapists would then be able to carry out cardiac rehabilitation for patients earlier, thus improving their postoperative prognosis. This might provide a reference value for recovery and early rehabilitation for cardiac surgery.

### Limitations

This study is the first to explore the association between grip recovery and postoperative complications after discharge among middle-aged and older adult people. Patients’ follow-up continued through 30 days after hospital discharge. Our study includes certain limitations. First, our research was a single-center study with a small number of people. A single location is not widely representative, so we will perform a multicenter study next. Despite our sample was small, it still demonstrated a relationship between grip recovery and post-discharge complications in cardiac surgery patients. Second, we did not distinguish between types of cardiac surgery. This kind of distinction is needed in follow-up studies. Third, the surgical prognosis needs to be observed for a relatively longer period of time.

## Conclusion

This pilot study demonstrates that grip recovery is a good way use of grip value for 30-day complication after discharge. It showed that poor grip recovery may relate to a higher risk of post discharge complications within 30 days after discharge in middle-aged and older people independent of surgical risk. The cutoff point was 83.29%, which might serve as a reference for predicting 30-day complications. We also found poor grip recovery might be associated with a longer hospital stay. The results of this study provide a reference for the development of rehabilitation programs in the early postoperative recovery period and may also serve as prognostic indicators for postoperative high-risk groups.

## Supplementary information

**Additional file 1.** Supplementary content of methods. Methods for gait function and grip strength and figure of handheld dynamometer.

## Data Availability

The datasets used and/or analysed during the current study are de-identified and available from the corresponding author on reasonable request.

## Abbreviations

6MWD: 6 min walk distance
AUC: Area under the receiver operating characteristic curve
BMI: Body mass index
CABG: Coronary artery bypass grafting
ICU: Intensive care unit
IPAQ: International physical activity questionnaire
OR: Odds ratio
ROC: Receiver-operating characteristic
