# Supplementary material for: Perioperative poor grip strength recovery is associated with 30-day complication rate after cardiac surgery discharge in middle-aged and older adults - a prospective observational study
Source: BMC Cardiovasc Disord. 2019 Nov 27;19:266. doi: 10.1186/s12872-019-1241-x (PMC6882174; doi:10.1186/s12872-019-1241-x)

Supplementary content

Methods for gait function and grip strength

Grip strength (kg) was used as a measure of muscle strength and was quantified using a handheld dynamometer (GRIP-D; Takei Ltd, Niigata, Japan). Participants were asked to exert their maximum effort twice using their dominant hand and the average grip strength was recorded. Gait function was assessed with the 4-m walk tests and the TUGT. To measure walking speed, two photocells connected to a recording chronometer were placed at the beginning and the end of a 4-meter course at the site clinic. Participants were instructed to stand with both feet touching the starting line and to begin walking at their usual pace after a verbal command was given. The time between activation of the first and the second photocell was measured and the average speed of two walks was recorded.

Figure of handheld dynamometer (GRIP-D; Takei Ltd, Niigata, Japan)


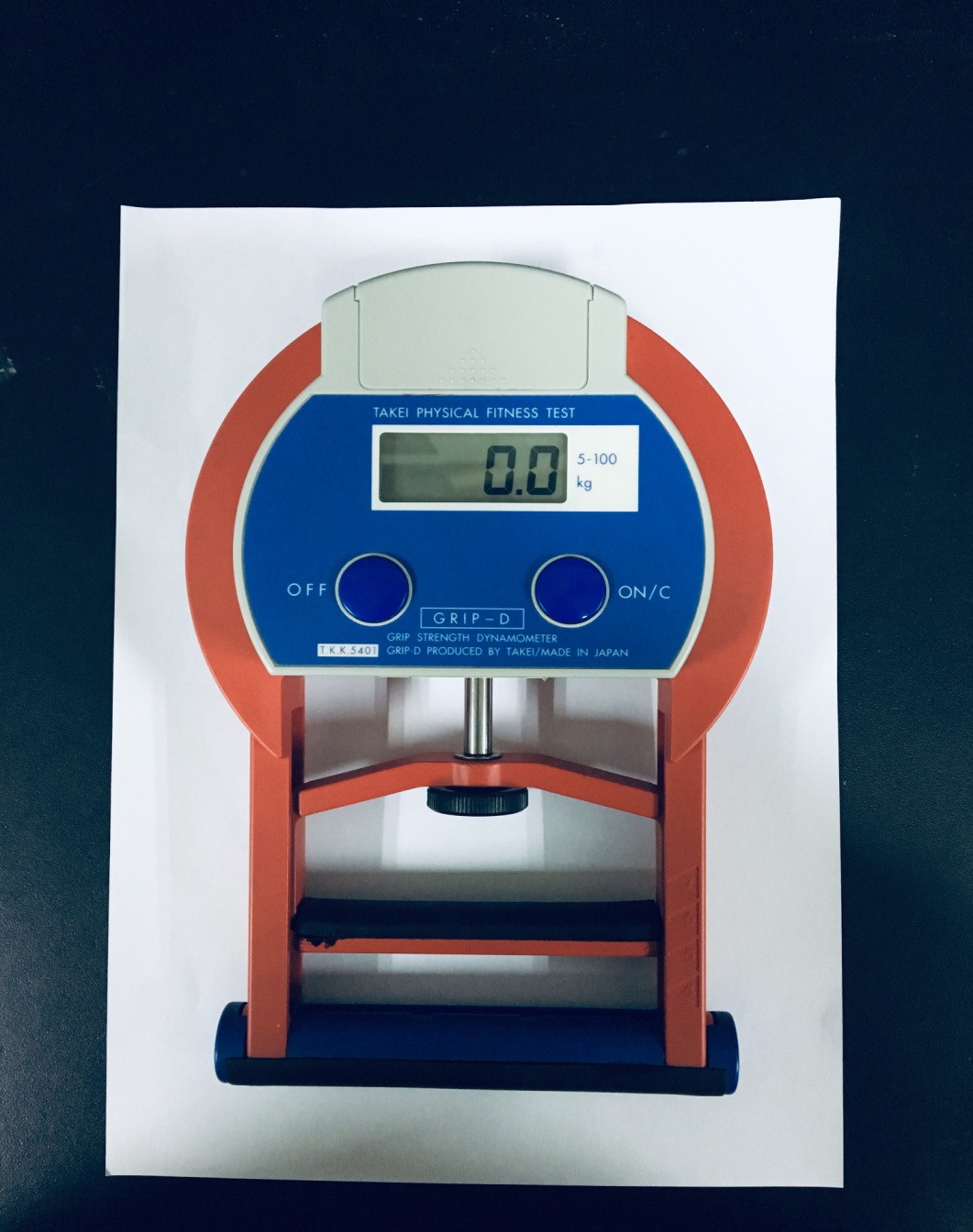

Supplement: Supplementary file 1 — Additional file 1. Supplementary content of methods. Methods for gait function and grip strength and figure of handheld dynamometer. [file 12872_2019_1241_MOESM1_ESM.docx]
